# Supplementary material for: Resolving the full spectrum of human genome variation using Linked-Reads
Source: Genome Res. 2019 Apr;29(4):635–45. doi: 10.1101/gr.234443.118 (PMC6442396; doi:10.1101/gr.234443.118)
Supplement: Supplemental Material [file supp_gr.234443.118_Supplemental_longranger-2.2.zip › longranger-2.2.tar.bz2!/lr2.2-public/lib/stan/cmdstan/stan/doc/api/html/seq__view_8hpp_source.html]

Stan: src/stan/math/seq\_view.hpp Source File


|  |  |
| --- | --- |
|  | Stan  2.5.0  probability, sampling & optimization |


- Main Page
- Related Pages
- Namespaces
- Classes
- Files

- File List
- File Members

All Classes Namespaces Files Functions Variables Typedefs Enumerations Enumerator Friends Macros Pages

- src
- stan
- math

seq\_view.hpp

Go to the documentation of this file.

1 #ifndef STAN\_\_MATH\_\_SEQ\_VIEW\_HPP

2 #define STAN\_\_MATH\_\_SEQ\_VIEW\_HPP

3

4 #include <vector>

5

6 #include <stan/math/matrix/Eigen.hpp>

7

8 namespace stan {

9

10  namespace math {

11

12

13  template <typename T>

14  struct store\_type {

15  typedef const T& type;

16  };

17  template <>

18  struct store\_type<double> {

19  typedef const double type;

20  };

21  template <>

22  struct store\_type<int> {

23  typedef const int type;

24  };

25

26

27  template <typename T>

28  struct pass\_type {

29  typedef const T& type;

30  };

31  template <>

32  struct pass\_type<double> {

33  typedef double type;

34  };

35  template <>

36  struct pass\_type<int> {

37  typedef int type;

38  };

39

40

41  // S assignable to T

42  template <typename T, typename S>

43  class seq\_view {

44  private:

45  typename store\_type<S>::type x\_;

46  public:

47  seq\_view(typename pass\_type<S>::type x)

48  : x\_(x) {

49  }

50  inline typename pass\_type<T>::type

51  operator[](int n) const {

52  return x\_;

53  }

54  int size() const {

55  return 1;

56  }

57  };

58

59  template <typename T, typename S>

60  class seq\_view<T, Eigen::Matrix<S,Eigen::Dynamic,1> > {

61  private:

62  typename store\_type<Eigen::Matrix<S,Eigen::Dynamic,1> >::type x\_;

63  public:

64  seq\_view(typename pass\_type<Eigen::Matrix<S,Eigen::Dynamic,1> >::type x)

65  : x\_(x) {

66  }

67  inline typename pass\_type<T>::type

68  operator[](int n) const {

69  return x\_(n);

70  }

71  int size() const {

72  return x\_.size();

73  }

74  };

75

76

77  template <typename T, typename S>

78  class seq\_view<T, Eigen::Matrix<S,1,Eigen::Dynamic> > {

79  private:

80  typename store\_type<Eigen::Matrix<S,1,Eigen::Dynamic> >::type x\_;

81  public:

82  seq\_view(typename pass\_type<Eigen::Matrix<S,1,Eigen::Dynamic> >::type x)

83  : x\_(x) {

84  }

85  inline typename pass\_type<T>::type

86  operator[](int n) const {

87  return x\_(n);

88  }

89  int size() const {

90  return x\_.size();

91  }

92  };

93

94

95

96  // row-major order of returns to match std::vector

97  template <typename T, typename S>

98  class seq\_view<T, Eigen::Matrix<S,Eigen::Dynamic,Eigen::Dynamic> > {

99  private:

100  typename store\_type<Eigen::Matrix<S,Eigen::Dynamic,Eigen::Dynamic> >::type x\_;

101  public:

102  seq\_view(typename pass\_type<Eigen::Matrix<S,Eigen::Dynamic,Eigen::Dynamic> >::type x)

103  : x\_(x) {

104  }

105  inline typename pass\_type<T>::type

106  operator[](int n) const {

107  return x\_(n / x\_.cols(), n % x\_.cols());

108  }

109  int size() const {

110  return x\_.size();

111  }

112  };

113

114  // question is how expensive the ctor is

115  template <typename T, typename S>

116  class seq\_view<T, std::vector<S> > {

117  private:

118  typename store\_type<std::vector<S> >::type x\_;

119  const size\_t elt\_size\_;

120  public:

121  seq\_view(typename pass\_type<std::vector<S> >::type x)

122  : x\_(x),

123  elt\_size\_(x\_.size() == 0 ? 0 : seq\_view<T,S>(x\_[0]).size()) {

124  }

125  inline typename pass\_type<T>::type

126  operator[](int n) const {

127  return seq\_view<T,S>(x\_[n / elt\_size\_])[n % elt\_size\_];

128  }

129  int size() const {

130  return x\_.size() \* elt\_size\_;

131  }

132  };

133

134  // BELOW HERE JUST FOR EFFICIENCY

135

136  template <typename T>

137  class seq\_view<T, std::vector<T> > {

138  private:

139  typename store\_type<std::vector<T> >::type x\_;

140  public:

141  seq\_view(typename pass\_type<std::vector<T> >::type x)

142  : x\_(x) {

143  }

144  inline typename pass\_type<T>::type

145  operator[](int n) const {

146  return x\_[n];

147  }

148  int size() const {

149  return x\_.size();

150  }

151  };

152

153  // if vector of S with S assignable to T, also works

154  // use enable\_if? (and disable\_if for the general case)

155  template <typename T>

156  class seq\_view<T, std::vector<std::vector<T> > > {

157  private:

158  typename store\_type<std::vector<std::vector<T> > >::type x\_;

159  const size\_t cols\_;

160  public:

161  seq\_view(typename pass\_type<std::vector<std::vector<T> > >::type x)

162  : x\_(x),

163  cols\_(x\_.size() == 0 ? 0 : x\_[0].size()) { }

164  inline typename pass\_type<T>::type

165  operator[](int n) const {

166  return x\_[n / cols\_][n % cols\_];

167  }

168  int size() const {

169  return x\_.size() \* cols\_;

170  }

171  };

172

173  template <>

174  class seq\_view<double, std::vector<int> > {

175  private:

176  store\_type<std::vector<int> >::type x\_;

177  public:

178  seq\_view(pass\_type<std::vector<int> >::type x)

179  : x\_(x) {

180  }

181  inline pass\_type<double>::type operator[](int n) const {

182  return x\_[n];

183  }

184  int size() const {

185  return x\_.size();

186  }

187  };

188

189

190

191

192  }

193 }

194

195 #endif

stan::math::seq\_view< T, Eigen::Matrix< S, Eigen::Dynamic, Eigen::Dynamic > >::size

int size() const

**Definition:** seq\_view.hpp:109

stan::math::seq\_view< T, std::vector< S > >::operator[]

pass\_type< T >::type operator[](int n) const

**Definition:** seq\_view.hpp:126

stan::math::seq\_view::size

int size() const

**Definition:** seq\_view.hpp:54

stan::math::seq\_view< T, Eigen::Matrix< S, 1, Eigen::Dynamic > >::size

int size() const

**Definition:** seq\_view.hpp:89

stan::math::seq\_view< T, std::vector< T > >::size

int size() const

**Definition:** seq\_view.hpp:148

stan::math::seq\_view< T, Eigen::Matrix< S, 1, Eigen::Dynamic > >::seq\_view

seq\_view(typename pass\_type< Eigen::Matrix< S, 1, Eigen::Dynamic > >::type x)

**Definition:** seq\_view.hpp:82

stan::math::seq\_view::seq\_view

seq\_view(typename pass\_type< S >::type x)

**Definition:** seq\_view.hpp:47

stan::math::seq\_view< T, std::vector< S > >::size

int size() const

**Definition:** seq\_view.hpp:129

stan::math::pass\_type< double >::type

double type

**Definition:** seq\_view.hpp:33

stan::math::seq\_view< T, Eigen::Matrix< S, 1, Eigen::Dynamic > >::operator[]

pass\_type< T >::type operator[](int n) const

**Definition:** seq\_view.hpp:86

stan::math::pass\_type< int >::type

int type

**Definition:** seq\_view.hpp:37

stan::math::seq\_view< T, Eigen::Matrix< S, Eigen::Dynamic, 1 > >::seq\_view

seq\_view(typename pass\_type< Eigen::Matrix< S, Eigen::Dynamic, 1 > >::type x)

**Definition:** seq\_view.hpp:64

stan::math::seq\_view< double, std::vector< int > >::size

int size() const

**Definition:** seq\_view.hpp:184

stan::math::pass\_type

**Definition:** seq\_view.hpp:28

stan::math::seq\_view< double, std::vector< int > >::operator[]

pass\_type< double >::type operator[](int n) const

**Definition:** seq\_view.hpp:181

stan::math::seq\_view< T, std::vector< S > >::seq\_view

seq\_view(typename pass\_type< std::vector< S > >::type x)

**Definition:** seq\_view.hpp:121

stan::math::seq\_view< T, Eigen::Matrix< S, Eigen::Dynamic, 1 > >::size

int size() const

**Definition:** seq\_view.hpp:71

stan::math::seq\_view< double, std::vector< int > >::seq\_view

seq\_view(pass\_type< std::vector< int > >::type x)

**Definition:** seq\_view.hpp:178

Eigen.hpp

stan::math::seq\_view< T, std::vector< std::vector< T > > >::size

int size() const

**Definition:** seq\_view.hpp:168

stan::math::seq\_view< T, std::vector< std::vector< T > > >::seq\_view

seq\_view(typename pass\_type< std::vector< std::vector< T > > >::type x)

**Definition:** seq\_view.hpp:161

stan::math::seq\_view< T, Eigen::Matrix< S, Eigen::Dynamic, Eigen::Dynamic > >::operator[]

pass\_type< T >::type operator[](int n) const

**Definition:** seq\_view.hpp:106

stan::math::seq\_view< T, std::vector< T > >::operator[]

pass\_type< T >::type operator[](int n) const

**Definition:** seq\_view.hpp:145

stan::math::seq\_view

**Definition:** seq\_view.hpp:43

stan::math::store\_type< int >::type

const int type

**Definition:** seq\_view.hpp:23

stan::math::pass\_type::type

const T & type

**Definition:** seq\_view.hpp:29

stan::math::seq\_view< T, Eigen::Matrix< S, Eigen::Dynamic, 1 > >::operator[]

pass\_type< T >::type operator[](int n) const

**Definition:** seq\_view.hpp:68

stan::math::seq\_view< T, std::vector< std::vector< T > > >::operator[]

pass\_type< T >::type operator[](int n) const

**Definition:** seq\_view.hpp:165

stan::math::seq\_view< T, Eigen::Matrix< S, Eigen::Dynamic, Eigen::Dynamic > >::seq\_view

seq\_view(typename pass\_type< Eigen::Matrix< S, Eigen::Dynamic, Eigen::Dynamic > >::type x)

**Definition:** seq\_view.hpp:102

stan::math::store\_type

**Definition:** seq\_view.hpp:14

stan::math::store\_type::type

const T & type

**Definition:** seq\_view.hpp:15

stan::math::store\_type< double >::type

const double type

**Definition:** seq\_view.hpp:19

stan::math::seq\_view< T, std::vector< T > >::seq\_view

seq\_view(typename pass\_type< std::vector< T > >::type x)

**Definition:** seq\_view.hpp:141

stan::math::seq\_view::operator[]

pass\_type< T >::type operator[](int n) const

**Definition:** seq\_view.hpp:51


---

[ Stan Home Page ]

*© 2011–2014,
Stan Development Team.*
